# SARS-CoV-2 antibody prevalence, correlates, and access to harm reduction services among people who inject drugs living with and without HIV and their partners in Kenya

**DOI:** 10.1186/s12954-023-00754-5

**Published:** 2023-02-23

**Authors:** Shradha Doshi, Hanley Kingston, Ashley S. Tseng, Bhavna H. Chohan, Betsy Sambai, Brandon L. Guthrie, Aliza Monroe-Wise, Loice W. Mbogo, Sarah Masyuko, Khai Hoan Tram, William Sinkele, Paul Macharia, David Bukusi, Joshua T. Herbeck, Carey Farquhar

**Affiliations:** 1 Aga Khan University Hospital, Nairobi, Kenya; 2 Department of Global Health, University of Washington, Seattle, WA USA; 3 Department of Epidemiology, University of Washington, Seattle, WA USA; 4 Kenyatta National Hospital, Nairobi, Kenya; 5 Ministry of Health, Nairobi, Kenya; 6 Kenya Medical Research Institute, Nairobi, Kenya; 7 University of Washington-Global Assistance Programme, Nairobi, Kenya; 8 Department of Medicine, University of Washington, Seattle, WA USA; 9 Support for Addiction Prevention and Treatment in Africa, Nairobi, Kenya

**Keywords:** SARS-CoV-2 antibody, HIV/AIDS, Sub-Saharan Africa, PWID, Kenya

## Abstract

**Background:**

In sub-Saharan Africa many people who inject drugs (PWID) are living with undiagnosed or untreated HIV and experience high levels of poverty and conditions that can contribute to worse outcomes from SARS-CoV-2 infection. Identifying the burden of SARS-CoV-2 infection in marginalized populations like PWID may contribute to controlling the pandemic.

**Methods:**

This is a nested cross-sectional study within an ongoing cohort study that recruits PWID living with HIV and their injecting and/or sexual partners at needle and syringe program sites and methadone clinics in Kenya. Blood samples were collected from consenting participants at enrollment to determine SARS-CoV-2 antibodies using a Platellia BioRad SARS-CoV-2 total antibody enzyme-linked immunosorbent assay. Baseline data were collected on HIV status, antiretroviral therapy and methadone adherence. We used logistic regression to identify factors associated with antibody positivity and descriptive statistics to report SARS-CoV-2 antibody prevalence.

**Results:**

One thousand participants were enrolled between April and July 2021, of whom 323 (32.3%) were women and 677 (67.7%) were men. Median age of participants was 36 years (interquartile range: 30, 42). SARS-CoV-2 antibody positivity was found in 309 (30.9%) participants. Disruption in obtaining methadone service was reported by 106 (24.3%) of the participants. Men were significantly less likely than women to have SARS-CoV-2 antibodies (adjusted odds ratio [aOR] = 0.68, 95% confidence interval [CI] 0.51, 0.95; *p* < 0.01) Participants who reported a sexual or injecting partner diagnosed with SARS-CoV-2 were twofold more likely to have SARS-CoV-2 antibodies detected (aOR = 2.21, 95% CI 1.06, 4.58; *p* < 0.032). Living with HIV was not associated with presence of SARS-CoV-2 antibodies.

**Conclusion:**

The seroprevalence of SARS-CoV-2 of 30.9% in this cohort suggests high transmission rates within this population. SARS-CoV-2 seroprevalence was similar for people living with and without HIV. A large portion of this population was noted to have had disruption in access to harm reduction services.

## Key findings

1. We found a high seroprevalence of SARS-CoV-2 antibodies in people who inject drugs and their sexual and injecting partners.
2. We also found that participants who reported a sexual or injecting partner diagnosed with SARS-CoV-2 were more likely to have SARS-C0V-2 antibody positivity.
3. Living with HIV was not associated with presence of SARS-CoV-2 antibodies.
4. A large proportion of people who inject drugs was noted to have had disruptions in access to harm reduction services during the study period.

**Key implications**

1. Kenya’s national healthcare policymakers should implement special interventions like increased access to ‘take-home doses’ of methadone and waivers for daily in-person check--ns for this population during current and future crises to maintain continuity of treatment and access to harm reduction services.

## Introduction

People who inject drugs (PWID) in sub-Saharan Africa are more likely than the general population to be living with undiagnosed HIV and to experience poverty, food and housing instability, stigma, and discrimination that could lead to increased transmission of SARS-CoV-2 and worse outcomes for those with the respiratory viral disease. It is also more challenging for PWID to access healthcare when services have been limited by lockdowns and to adhere to social distancing measures to prevent spread of respiratory viruses, such as SARS-CoV-2 that causes COVID-19 [1]. In addition, recent emergence of newer SARS-CoV-2 variants has led to multiple waves of outbreaks which threaten to halt the progress made thus far in the spread of the virus.

In Kenya, from January 2020 to December 2021, there were more than 300,000 confirmed cases of SARS-CoV-2 infections and over 5400 reported deaths [2]. Poorly controlled HIV infection, when combined with socio-economic vulnerability and co-morbid conditions associated with injection drug use, may result in increased SARS-CoV-2 acquisition and transmission, more symptomatic COVID-19 disease [3], and poor clinical outcomes [4–7]. While cohort studies and meta-analyses from the USA and Spain who have relatively low numbers of people living with HIV (PLWH) did not find current HIV infection to be a risk factor for SARS-COV-2 infections [8, 9], larger population-level cohort studies have found PLWH to have a higher risk for COVID-19 associated morbidity [4, 6, 10–12]. Despite this, little COVID-19 testing is being conducted among PWID and their social networks in sub-Saharan Africa, limiting understanding of the pandemic’s effects on their health and well-being. Determining SARS-COV-2 antibody prevalence, defining transmission dynamics of SARS-CoV-2, and its risk factors in this study population will provide foundational data to inform Kenyan national strategies around access to HIV and methadone services and potentially scale-up COVID-19 testing, vaccination, surveillance and other public health measures for PWID.

## Methods

### Study design

This cross-sectional study was nested within an ongoing prospective cohort study. From April – July 2021 we recruited 1000 PWID and their sexual and injecting partners, 500 living with HIV and 500 living without HIV. All were participants in the parent cohort study and were recruited at needle-syringe program (NSP) sites and methadone clinics in Kenya. To conduct the study, we partnered with 4 non-governmental organizations: Support for Addictions Prevention and Treatment in Africa for Nairobi sites; Omari Project and MEWA in Kilifi county; and Reachout Centre in Mombasa county.

### Study population

Participants were men and women above the age of 18 years who were enrolled in a study of HIV and hepatitis C-assisted partner services among PWID (NIH R01DA043409) which has been described in detail elsewhere [13]. All participants had either injected drugs within the last year or been named as a sexual or injecting partner of a PLWH who had injected drugs within the last year.

### Clinical procedures

#### Screening and recruitment

Participants were informed of the study when they came to the NSP for services or were invited by peer educators when they went into the community to distribute harm reduction messages, needles, syringes, and condoms, and to find PWID who were due for their HIV care. The individuals who agreed to participate in the study were asked to sign a written consent form and were subsequently enrolled in the study.

#### Enrollment

Study staff assigned unique participant identification numbers using a biometric iris scanner to prevent duplicate enrollment. Baseline data were collected via a questionnaire asking about socio-demographic characteristics, HIV status, drug use, methadone adherence, and behaviors that could increase risk of SARS-CoV-2 infection. Current and recent past medical history was obtained to capture any clinical symptoms or adverse outcomes that they may have experienced due to SARS-CoV-2 infection. Approximately 10 ml of blood was collected in an EDTA vacutainer from all participants at enrollment for SARS-CoV-2 antibody testing.

### Laboratory procedures

Blood samples from recruitment sites in the Coast region were transported daily to Malindi Sub-county hospital laboratory in cooler boxes, processed and stored in -80 °C freezers, and shipped weekly in dry ice to the University of Nairobi laboratory. Samples from the Nairobi region were shipped directly in cooler boxes to the University of Nairobi laboratory where they were processed, separated into aliquots, frozen, and serological immunoassays were performed on all the samples. Samples were tested for detection of total antibodies SARS-CoV-2 to SARS-CoV-2 nucleocapsid protein using a Platellia SARS-CoV-2 total antibody enzyme-linked immunosorbent assay (ELISA) (Bio-Rad, Marnes-la-Coquette, France). This qualitative SARS-CoV-2 ELISA serology assay, which had United States Food and Drug Administration Emergency Use Authorization, detects total (IgM/IgA/IgG) antibodies to the nucleocapsid protein of SARS-CoV-2 in plasma with reported 94.9% specificity and 97.4% sensitivity [14]. The result of the serology assay was based on the ratio of the optical density (OD) value of the sample to the mean OD of the test kit cut-off controls. The sample was considered positive for SARS-CoV-2 antibodies if the OD ratio was ≥ 1.0, negative if < 0.8, and equivocal if ≥ 0.8 and < 1.0, according to the kit insert. Samples that were equivocal were retested before final interpretation. The laboratory was enrolled in External Quality Control program and had performed well in the quality assessment for the SARS-CoV-2 Platellia ELISA assay during the period of the sample testing.

During the period of our study, only the ChAdOx1 (*Oxford/AstraZeneca*) COVID-19 vaccine was available in Kenya. The Oxford/AstraZeneca ChAdOx1 vaccine induces an anti-spike antibody response. The Platellia SARS-CoV-2 total antibody assay to nucleocapsid protein yields a negative result for participants who have been vaccinated with the Oxford/AstraZeneca ChAdOx1 vaccine, thus allowing us to differentiate between people who were exposed to the virus from those not exposed, regardless of COVID-19 vaccination status.

### Statistical analyses

The study population was characterized in terms of demographic characteristics, HIV status, SARS-CoV-2 exposures, and experiences with methadone treatment, antiretroviral therapy (ART) services, and pre-exposure prophylaxis (PrEP) services during the 3 months prior to enrollment. All variables were treated as categorical and summarized as counts and proportions. For each demographic characteristic, the proportion of individuals in each stratum who tested antibody-positive was calculated. Univariate logistic regression was used to identify demographic factors associated with antibody positivity. All factors from univariate logistic regression with *p* < 0.20 (sex, age, relationship status, region of enrollment, taking methadone, and having a sexual or injecting partner diagnosed with SARS-CoV-2), were included in a multivariate logistic regression model. A *p*-value < 0.05 was interpreted as statistically significant in either analysis.

### Consent and ethical approval

The proposal was presented to and approved by the Institutional Review Board at the University of Washington, Seattle, and the Ethics and Research Committee at Kenyatta National Hospital/University of Nairobi. Participants were screened for eligibility into the study. The study was clearly explained to potential participants in their preferred language (Kiswahili or English), and written informed consent was obtained prior to enrollment.

## Results

### Participant characteristics

A total of 1000 participants were enrolled in the study between April and July 2021, of whom 323 (32.3%) were women and 677 (67.7%) were men. Overall, 228 (70.6%) of the 323 women were living with HIV and 272 (40.2%) of the 677 men were living with HIV. Median age of the participants was 36 years (Interquartile range 30–42). A minority of participants (29.8%) were married or partnered and 59.3% reported primary level of education or less (< 8 years). The majority of participants (810 [81%]) reported having injected drugs in the past week. Almost equal proportions of HIV positive and HIV negative participants were noted to have injected drugs in the past 1 week (401 [80.2%] vs. 409 [81.8%], respectively). Current use of methadone was reported by 43.6% of participants and 88.9% reported stable housing conditions (Table 1).

**Table 1:** Differences in demographic characteristics by HIV status for persons who inject drugs and their sexual and injecting partners (*N* = 1000)

	Total (*N* = 1000)	Living with HIV (*N* = 500)	Not living with HIV (*N* = 500)
	*n* (%) or median (IQR)		
*Sex*			
Female	323 (32.3)	228 (45.6)	95 (19.0)
Male	677 (67.7)	272 (54.4)	405 (81.0)
*Age (years)*			
Median (IQR)	36.0 (30.0,42.0)	38.0 (32.8–44.0)	34.0 (28.0, 39.0)
18–35	473	182 (36.4)	291 (58.2)
36–74	527	318 (63.6)	209 (41.8)
*Relationship Status*			
Single	362 (36.2)	167 (33.4)	195 (39.0)
Married or partnered	298 (29.8)	149 (29.8)	149 (29.8)
Divorced or separated	340 (34.0)	184 (36.8)	156 (31.2)
*Education Level*			
No formal education	61(0.6)	40 (8.0)	21 (4.2)
Primary level	593 (59.3)	305 (61.0)	288 (57.6)
Secondary level	306 (30.6)	145 (29.0)	161 (32.2)
Tertiary level	40 (0.4)	10 (2.0)	30 (15.0)
*Injected in the Past Week*			
Yes	810 (81.0)	401 (80.2)	409 (81.8)
No	190 (19.0)	99 (19.8)	91 (18.2)
*Currently Taking Methadone*			
Yes	436 (43.6)	187 (37.4)	249 (49.8)
No	564 (56.4)	313 (62.6)	251 (50.2)
*Reported Stable Housing*^*1*^			
Yes	889 (88.9)	448 (89.6)	441 (88,2)
No	109 (10.9)	52 (10.4)	57 (11.4)

^1^Two participants had missing data

### SARS-CoV-2 antibody prevalence and correlates among PWID and their sexual or injecting partners

Among 1000 participants, SARS-CoV-2 total antibody was detected in 309 (30.9%) (679 tested negative and 12 tests were equivocal). Of the 309 participants who tested positive for SARS-CoV-2 antibodies, 187 (60.5%) reported symptoms consistent with COVID-19 during the last 3 months, including cough, fever and muscle aches. (Table 3). This proportion was no different than the proportion reporting COVID-like symptoms among those who were antibody negative (60.5% vs. 61.2%; *p* = 0.82). None of the participants reported having been diagnosed with SARS-CoV-2 or being hospitalized for their symptoms. In adjusted multivariate analysis, men were significantly less likely than women to have SARS-CoV-2 antibodies (aOR = 0.68, 95% confidence interval [CI] 0.51, 0.95; *p* = 0.01) and participants from the Coast region had lower odds of SARS-CoV-2 antibody positivity compared to the Nairobi region (aOR = 0.72, 95% CI, 0.54, 0.95; *p* = 0.022) (Table 2).

**Table 2:** Characteristics associated with SARS-CoV-2 antibody prevalence in persons who inject drugs and their partners

Characteristic	Number of total participants (*N* = 309)	Percentage SARS-CoV-2 antibody positive	Odds Ratio (OR) (95% CI)	*P*-value	Adjusted OR (aOR) (95% CI)*	*P*-value
*Sex*						
Female	119	37.2	Ref	Ref	Ref	Ref
Male	190	28.4	0.67 (0.59, 0.91)	0.006	0.68 (0.51–0.95)	0.010
*Age (years)*						
18–35	135	29.0	Ref	Ref	Ref	Ref
36–74	174	33.3	1.23 (0.94, 1.61)	0.14	1.23 (0.93–1.64)	0.153
*Relationship Status*						
Single	99	27.7	Ref	Ref	Ref	Ref
Married or partnered	87	29.5	1.09 (0.77, 1.53)	0.620	1.14 (0.80–1.62)	0.48
Divorced or separated	123	36.7	1.50 (1.09, 2.08)	0.013	1.47 (1.05–2.06)	0.024
*Education Level*						
No formal education	20	32.8	Ref	Ref		
Primary level	177	30.3	0.89 (0.51, 1.59)	0.683		
Secondary level	96	31.7	0.95 (0.53, 1.74)	0.866		
Tertiary level	16	41.0	1.43 (0.67, 3.29)	0.403		
*Region of Enrollment*						
Nairobi	171	34.7	Ref	Ref	Ref	Ref
Coast	138	27.9	0.73 (0.55, 0.95)	0.021	0.72 (0.54, 0.95)	0.022
*HIV Status*						
Living with HIV	151	30.6	0.93 (0.72, 1.23)	0.631		
Living without HIV	158	32.0	Ref	Ref		
*Injected in the Past Week*						
Yes	250	31.2	0.98 (0.70, 1.39)	0.928		
No	59	31.6	Ref	Ref		
*Currently Taking Methadone*						
Yes	124	28.8	0.81 (0.62, 1.07)	0.135	0.91(0.68–1.21)	0.506
No	185	32.8	Ref	Ref	Ref	Ref
*High-Risk Exposure in Past 2 Weeks*						
Yes	9	31.0	0.99 (0.42, 2.14)	0.977		
No	300	31.3	Ref	Ref		
*Any Sexual or Injecting Partners Diagnosed with COVID-19*						
Yes	15	46.9	1.99 (0.97, 4.04)	0.057	2.21 (1.06–4.58)	0.032
No	294	30.8	Ref	Ref	Ref	Ref
*Any Housing Partners Diagnosed with COVID-19*						
Yes	8	23.5	0.67 (0.28, 1.43)	0.325		
No	301	31.6	Ref	Ref		

*CI* confidence interval

*Includes all variables associated with non-suppression in univariate analysis at *p* < 0.20

Participants who had a sexual or injecting partner diagnosed with were twice as likely to have SARS-CoV-2 antibodies detected compared to those without a partner reporting a COVID-19 diagnosis (aOR = 2.21, 95% CI, 1.06, 4.58; *p* = 0.032). Those who were divorced or separated were significantly more likely to have SARS-CoV-2 antibodies, compared to those who were single (aOR = 1.47, 95% CI 1.05, 2.06, *p* = 0.024). Living with HIV was not significantly associated with presence of SARS-CoV-2 antibodies (aOR = 0.93, 95% CI 0.72, 1.23, *p* = 0.63). Age, housing instability, current injection use, and current methadone use were also not significantly associated with presence of SARS-CoV-2 antibodies.

### Reports of COVID-19 exposures

Only 29 (2.9%) participants reported a high-risk exposure to SARS-CoV-2 virus in the past 2 weeks, defined as being in close physical contact (6 feet or closer for at least 15 min) with someone who had recently tested positive for COVID-19 or had any symptoms consistent with COVID-19 (Table 3). Thirty-two (3.2%) participants reported that a sexual or injecting partner had been diagnosed with COVID-19 and 44 (4.4%) reported that a sexual or injecting partner had died from COVID-19 or a flu-like illness since March 2020. Thirty-four (3.4%) of the participants reported that someone they lived with had been diagnosed with SARS-CoV-2 virus. Thirty (3%) of the participants reported that someone they lived with had died due to SARS-CoV-2 or flu-like illness since March 2020.

**Table 3:** SARS-CoV-2 antibody positivity and reported COVID-19 exposures in persons who inject drugs and their partners, living with and without HIV (*N* = 1000)

Characteristic	All (*N* = 1000)	SARS-CoV-2 seropositive (*N* = 309)
	*n* (%)	
COVID-19 Symptoms in Past 2 Weeks*		
Fever or chills	231 (23.1)	65 (21.0)
Cough	305 (30.5)	90 (29.1)
Shortness of breath or difficulty breathing	92 (9.2)	25 (8.1)
Muscle or body aches	179 (17.9)	46 (14.9)
Headache	250 (25.0)	79 (25.6)
New loss of taste or smell	56 (5.6)	17 (5.5)
Sore throat	88 (8.8)	20 (6.5)
Congestion or runny nose	261 (26.1%)	76 (24.6)
Nausea or vomiting	111 (11.1)	26 (8.4)
None	390 (39.0)	122 (39.5)
*High-Risk Exposure in Past 2 Weeks*		
Yes	29 (2.9)	9 (2.9)
No	971 (97.1)	300 (97.1)
*Any Sexual or Injecting Partners Diagnosed with COVID-19*		
Yes	32 (3.2)	15 (4.9)
No	968 (96.8)	294 (95.1)
*Any Sexual or Injecting Partners Died From COVID-19 or Flu-Like Illness since March 2020*		
Yes	44 (4.4)	15 (4.9)
No	956 (95.6)	294 (95.1)
*Any Housing Partners Diagnosed with COVID-19*		
Yes	34 (3.4)	8 (2.6)
No	966 (96.6)	301 (97.4)
*Any Housing Partners Died From COVID-19 or Flu-Like Illness since March 2020*		
Yes	30 (3.0)	7 (2.3)
No	969 (96.9)	301 (97.4)

*Participants with multiple symptoms are represented more than once

### Methadone program disruptions related to COVID-19 pandemic restrictions

Of the 436 (43.6%) participants who were currently enrolled in a methadone program, 106 (24.3%) reported problems obtaining methadone. Of the 106 participants, 22 (20.8%) reported that the problems related to obtaining methadone resulted from the COVID-19 pandemic restrictions.

### Antiretroviral therapy service disruptions due to the COVID-19 pandemic

Among the 500 participants living with HIV (228 women and 272 men), 485 (97%) reported to be currently on ART. Problems obtaining ART were experienced by 62 (12.4%) of the 485 participants of which 12 (19.3%) reported that the problems were directly related to the COVID-19 pandemic. Fifteen (3%) of the 500 participants who were not living with HIV reported PrEP use; however, no data were available on disruption of PrEP services.

### COVID-19 vaccination status

The last 331 participants enrolled were asked about their COVID-19 vaccination history. COVID-19 vaccines started becoming available to the general public after study enrollment began. Only 5 of the 331 (1.5%) persons interviewed reported having received the ChAdOx1 (Oxford/AstraZeneca) SARS-CoV-2 vaccine between April and July 2021.

## Discussion

This is among the first studies in Kenya to define seroprevalence of SARS-CoV-2 antibodies among PWID and their sexual or injecting partners. We found SARS-CoV-2 antibody prevalence between April and July 2021 to be high at 30.9%. Seroprevalence studies done among Kenyan blood donors (aged 16–64 years) from April to June 2020 were estimated at 4.3% [15] and 9.1% in August–September 2020 [16]. A general trend of rise in seropositivity with time since detection of the first confirmed COVID-19 case on March 13, 2020 has been noted in the few studies done in Kenya. In November 2020, 8 months after detection of the first case in Kenya, SARS-CoV-2 seropositivity in Nairobi was found to be 34.7% in a household-based study by Ngere et al. [17]. This study is one of a handful of population-based seroprevalence studies that have been conducted in Kenya and it selected households in each of Nairobi’s 17 sub-counties. However, similar to many studies in sub-Saharan Africa [15, 16, 18], it focused on the general populations and not members of key populations.

Globally, reports in the literature of SARS-CoV-2 antibody prevalence among PWID are also sparse. In a study of needle exchange clients in Stockholm, Sweden, between June and October 2020, SARS-CoV-2 antibody prevalence was 5.4% [19]. Between October 2020 and June 2021, participants from San Diego, California, USA and Tijuana, Baja California, Mexico who injected drugs were tested for SARS-CoV-2 antibodies and the seroprevalence was 36.3% (95% CI: 31.5, 41.1). These differences may be explained in part by the different lockdown measures in the different countries and the timelines during which the studies were conducted. Kenya has had four surges of the SARS-CoV-2 virus. The first surge began in June 2020 and plateaued in October 2020. The second surge peaked in the month of December 2020 while the third surge of cases started in March 2021 up to a plateau in June –July 2021 [20], and a fourth surge in December 2021 to January 2022. Our study was conducted 15–18 months after the first case was identified, and data collection began during an ongoing third surge, and at a time when vaccines were not yet widely available in Kenya.

Consistent with other studies conducted in Kenya [15, 16, 21, 22], we found significant differences in seroprevalence by region. In our study, participants from the Coast had lower odds for testing positive for the SARS-CoV-2 antibodies. These findings are in accordance with general population-based studies (among blood donors) that reported an antibody prevalence of 17.1% and 21.5% from participants tested between May and September 2020 in Mombasa (on the Coast of Kenya) and Nairobi respectively [23]. International points of entry are important channels of SARS-CoV-2 introduction into Kenya. The higher level of global and local interconnectivity in Nairobi makes it particularly vulnerable to the spread of the SARS-CoV-2 virus. Nairobi has higher population density and greater economic activities compared to the Coast region, both drivers of viral transmission.

In our study, living with HIV was not significantly associated with presence of SARS-CoV-2 antibodies. This was similarly noted in a study conducted in Western Kenya between January and March 2020 in which SARS-CoV-2 antibody positivity was reported as low as 3.1% in PLWH versus 4% in those without HIV (*p* = 0.68) [18]. In contrast, with regards to severe illness caused by SARS-CoV-2, the WHO Global Clinical Platform for COVID-19 produced a report on data from 37 countries showing that HIV infection is a significant independent risk factor for both severe or critical COVID-19 presentation at hospital admission and in-hospital mortality. Also noted was that the risk of developing severe or fatal COVID-19 was 30% greater in PLWH compared to people without HIV infection [24].

We also looked at disruptions in harm reduction services because this may lead to higher risks of overdose deaths and incident HIV and other infectious complications [25, 26]. Injecting alone to comply with social distancing requirements has also been recognized as a risk factor for opioid overdose [27]. Approximately a quarter of the participants reported problems accessing methadone during the study period, with 20.8% attribution these problems to COVID-19 related restrictions. Such restrictions might include travel and curfews, closure of harm reduction clinics or fear of contracting the SARS-CoV-2 virus from healthcare settings. The relatively high occurrence of disruption is in contrast to a study done in Sweden at the beginning of the pandemic where distribution of naloxone from needle exchange programmes was higher than the pre-pandemic period [19].

Numerous economic challenges also emerged during the pandemic and lack of funds to travel to clinics may have contributed to problems accessing methadone. Food programmes at NSP were also disrupted which may have been a lost incentive to come to the NSP. Furthermore, previous experience with drug-use stigmatization in health-care settings and cost of RT-PCR testing for SARS-CoV-2 may influence the PWID’s willingness to get tested or seek care for COVID-19 which is imperative to controlling the spread of the virus. Many PWID attend NSP centers and spend a large part of their day at these sites because they not only provide safer drug use supplies and counseling, but they also play an important role in their daily social life. Increased marginalization of this population may lead to increased drug use due to loss of their support system.

We were able to ask a subset of participants about their COVID-19 vaccination status. At the time of our study (April-July 2021), 5.5% of Kenyans had received at least one dose of the SARS-CoV-2 vaccine. In comparison, only 1.5% of our cohort reported having received at least one dose of the vaccine. NSPs constitute ideal settings to provide health interventions to PWID who generally access regular health care to a lesser extent than the population at large. Administration of vaccines within harm reduction services has been highly successful in other countries [28–30] and PWID in Kenya might also greatly benefit from provision of COVID-19 vaccines at NSP sites.

Our study is not without limitations and one of these is spike-based assay is more sensitive for detection of SARS-CoV-2 antibodies in early infection. Using serology assay for detection of both nucleocapsid and spike antibodies to SARS-CoV-2 could have increased our test sensitivity; however, we were limited during the pandemic to what was available at that time in the country. While a SARS-CoV-2 antibody prevalence of 30.9% appears high, another limitation was the lack of a comparison group in the general population. However, we were able to determine that there was no difference between those with and without HIV, one of our primary objectives. We were also able to determine that there was no difference in SARS-CoV-2 prevalence among those who reported injecting drugs versus those who did not. In conclusion, high SARS-CoV-2 antibody prevalence was noted in this population of PWID and their sexual and injecting partners; however, there is little COVID-19 related data and few studies that focus on the needs and risk profile of this key population. PWID are vulnerable and marginalized in most parts of the world and interventions to prevent disruption of services may need to be tailored to this population. Policy changes such as increased access to ‘take-home doses’ of methadone and temporarily waiving the daily in-person visit requirement may have helped ensure continuity of treatment.

In conclusion, the COVID-19 pandemic qualifies as what may be called a ‘big event’: a social crisis triggered by diseases, natural disasters, or financial crises that negatively affects PWID [31]. Beyond the COVID-19 pandemic, this key population is highly susceptible to emerging infectious diseases. They should be prioritized, and may benefit from targeted approaches when it comes to surveillance and prevention interventions.

## Declarations

### Study design

This is a nested cross-sectional study within an ongoing prospective cohort study.
